# Host-Directed Drug Therapies for Neglected Tropical Diseases Caused by Protozoan Parasites

**DOI:** 10.3389/fmicb.2018.02655

**Published:** 2018-11-30

**Authors:** Sanjay Varikuti, Bijay Kumar Jha, Greta Volpedo, Nathan M. Ryan, Gregory Halsey, Omar M. Hamza, Bradford S. McGwire, Abhay R. Satoskar

**Affiliations:** ^1^Department of Pathology, Wexner Medical Center, The Ohio State University, Columbus, OH, United States; ^2^Division of Infectious Diseases, Department of Internal Medicine, The Ohio State University, Columbus, OH, United States; ^3^Department of Microbiology, The Ohio State University, Columbus, OH, United States

**Keywords:** leishmaniasis, Chagas disease, Human African Trypanosomiasis, host-directed therapy, treatment

## Abstract

The neglected tropical diseases (NTDs) caused by protozoan parasites are responsible for significant morbidity and mortality worldwide. Current treatments using anti-parasitic drugs are toxic and prolonged with poor patient compliance. In addition, emergence of drug-resistant parasites is increasing worldwide. Hence, there is a need for safer and better therapeutics for these infections. Host-directed therapy using drugs that target host pathways required for pathogen survival or its clearance is a promising approach for treating infections. This review will give a summary of the current status and advances of host-targeted therapies for treating NTDs caused by protozoa.

## Introduction to Neglected Tropical Diseases

The neglected tropical diseases (NTDs) comprise a group including 20 different illnesses which currently affect over a billion individuals and amount to approximately 12% of the total global health burden across 149 tropical and subtropical countries (Chappuis et al., 2007; Ready, 2014; Mello et al., 2017). In humans, NTDs impair cognitive and physical development, cause development of chronic physical or emotional conditions, and could result in an increased mortality and morbidity having a significant economic impact on the economy in developing countries (Centers for Disease Control and Prevention, 2017; World Health Organization, 2018).

In this review we focus on three NTDs caused by different but related to protozoa which account for the highest death toll amongst all NTDs (Hotez et al., 2007): Leishmaniasis, caused by multiple species of the *Leishmania*; Chagas disease, caused by *Trypanosoma cruzi*; and Human African trypanosomiasis (HAT), caused by either *Trypanosoma brucei gambiense* or *Trypanosoma brucei rhodesiense*. According to the World Health Organization (WHO), these three NTDs require “Innovative and Intensified Disease Management (IDM)” approaches due to lacking research and development investments as well as a deficiency of effective control tools in endemic areas (Hotez et al., 2007).

Many NTDs are not a major concern in the most developed countries, but they continue to persist in areas where people live with poor sanitation and hygiene and remain in close contact with insect vectors and infected reservoir hosts. Tourists and military personnel traveling to endemic areas are also exposed to these infections and pose a risk of contracting them. Reports show that hundreds of US troops deployed in endemic areas such as Iraq have contracted cutaneous or visceral leishmaniasis (Weina et al., 2004). Additionally, immigration and increased exchanges of economic and social nature between different countries have contributed to the globalization of some NTDs such as Chagas disease (Kalil-Filho, 2015).

*Leishmania* and *Trypanosoma* not only infect humans but they also infect wild and domesticated animals, which serve as reservoirs for these diseases. Carnivores, rodents and lagomorphs have been identified as reservoirs for leishmaniasis in Europe, but the dog remains the main domestic reservoir, especially for *L. infantum* (Millan et al., 2014). Relocated or traveling dogs have been shown to bring *L. infantum* to non-endemic areas, spreading the disease throughout Europe (Maia and Cardoso, 2015). Along with the Mediterranean area, zoonotic leishmaniasis can be found in the Middle East, West Africa, Central Asia and the Americas. Here again wild animals and dogs are mainly responsible for *L. infantum* transmission (Harhay et al., 2011). Similarly, *Trypanosoma* can also infect a wide variety of domesticated and wild animals. For example, the presence of a widespread *T. cruzi* infection has been reported along the Texas-Mexico border in government working dogs (Meyers et al., 2017). In Uganda, the spread of sleeping sickness caused by *T. brucei rhodesiense* has been traced to infected cattle movement (Selby et al., 2013). Additionally, cases of horses infected with *T. evansi*, causative agent of the chronic wasting disease surra, have been reported in Brazil and other areas (Elshafie et al., 2013; Parreira et al., 2016). The widespread infection of livestock and other animals increases the risk of transmission to humans, severely impacting whole regions with the potential for global effect. Furthermore, animal infection can lead to infertility and loss of livestock resulting significant economic losses in many African, Asian and American countries (Giordani et al., 2016).

Despite the high prevalence of these three diseases, currently only a handful of treatments available against these parasites and many of those exhibit high toxicity due to the biomolecular similarities between eukaryotic parasites and mammalian cells, as well as to the accumulation of toxic derivative products of the therapeutic compounds. For instance, it is known that the toxicity of benznidazole and nifurtimox, established drugs for Chagas disease, is due to the metabolic conversion occurring after enzymatic reduction of nitro-groups (Bermudez et al., 2016). Another problem associated with some of these drugs is the increasing parasitic resistance as a result of adaptation. Resistance can arise due to different mechanisms such as target modifications, decreased drug uptake or increased efflux and augmented drug metabolism (Yasinzai et al., 2013). Several of these mechanisms, and a combination of them, have been documented in *Leishmania* parasites resistant to antimonials (Sb^III^, trivalent form reduced from the pentavalent form), miltefosine and amphotericin B (Ponte-Sucre et al., 2017). For example, reduced expression of the Sb^III^ transporter AQP1 leads to increased resistance to antimonials. Additionally, the uptaken Sb^III^ can be bound by the thiol compound trypanothione, present in *Leishmania*, and either sequestered or expelled from the cell via specific efflux pumps (Ponte-Sucre et al., 2017). Furthermore, increased expression of ABCG-like transporter TcABCG1 in *T. cruzi* confers resistance to benznidazole (Zingales et al., 2015).

Because of these concerns, it is imperative to find new therapeutics with low toxicity for the human host while maintaining high anti-parasitic efficacy. This review focuses on host-targeted approaches to treat NTDs caused by these three protozoan parasites.

## Host-Targeted Therapeutics

Host-targeted drugs bypass many of the problems encountered by treatments that specifically target parasites, by acting directly on host molecules or pathways that are redundant for the host but critical for pathogen invasion, survival and replication. Such approaches are likely to have a less chance of developing resistance as the host molecules and processes mutate at lower rates than most pathogens. Additionally, because these drugs act on the host, and not on specific pathogens, these treatments may be broad-spectrum and effective against several pathogens.

Different strategies have been employed to identify new host targets. The broader and more general approaches are transcriptomic and proteomic analysis as well as the assessment of microRNA, small interfering RNA (siRNA) and short hairpin RNA expression profiles (Prudencio and Mota, 2013; Krishnan and Garcia-Blanco, 2014). RNA interference in *Drosophila* has been previously used to identify several host factors manipulated by pathogens to their own advantage. This method was adopted to identify the host factors important for resistance to *Listeria monocytogenes* and *Chlamydia caviae* (Prudencio and Mota, 2013). Functional genomics have also been used to study gain or loss of function by over-expressing cDNA or iRNA respectively in mammalian cells to investigate the effects of different phenotypes on pathogenesis of intracellular pathogens. Additionally, hybrid interaction screens can be used to study protein-protein interaction between the host and the pathogen and can help identify potential host targets for drug therapy. Another method used to identify protein-protein as well as protein-RNA interactions is affinity chromatography (Krishnan and Garcia-Blanco, 2014).

Recent studies have identified several host-targeted therapeutics which show promise as novel drugs for treating neglected tropical parasitic infections. These approaches include the use of immuno-modulators, kinase inhibitors, and also natural compounds, which activate pro-inflammatory transcription factors like NF-Kb. Of these treatments, immuno-modulators are promising therapeutics not only used by themselves but also in combination with other drugs (Tiuman et al., 2011). In this review we focus on the host-targeted therapy and possible approaches to treat *Leishmania, T. brucei*, and *T. cruzi* infections.

## Leishmaniasis

Leishmaniasis is a group of tropical diseases caused by protozoan parasites of the genus *Leishmania* and transmitted via the bite of female Phlebotomine sandflies. This disease affects approximately 12 million people in more than 80 tropical and subtropical countries with incidence of reported cases rising rapidly at 2 million cases annually (McGwire and Satoskar, 2014). There are more than 20 different species of *Leishmania* distributed in both the Old and New World (Centers for Disease Control and Prevention, 2013; World Health Organization, 2018). The clinical manifestations of leishmaniasis depend on many factors including, interactions between the infecting *Leishmania* species and the immune response of the host, localized or disseminated parasite infection, genetic profile of the parasite and mammalian host, stress and also nutritional status of the host (Locksley et al., 1999; McCall et al., 2013). Cutaneous leishmaniasis (CL) is the most common form of leishmaniasis, which manifests as localized skin lesions that can become chronic, leading to significant tissue destruction and disfigurement. Other forms of infections are mucosal leishmaniasis (ML), or life-threatening visceral leishmaniasis (VL), which is the second most fatal parasitic infection after malaria and is characterized by dissemination of the parasites to liver, spleen and bone marrow ( The Center for Food Security and Public Health, 2017).

Although macrophages serve as the preferred host cell for *Leishmania*, this parasite can also infect other cells like dendritic cells (Woelbing et al., 2006; Contreras et al., 2014), mast cells (Bidri et al., 1997; Lu and Huang, 2017), fibroblasts (Hespanhol et al., 2005) and neutrophils (Mougneau et al., 2011). In particular, neutrophils are recruited within hours of the infection and delay the recruitment of dendritic cells, important for antigen presentation (Hurrell et al., 2015). Neutrophils seem to play an ambivalent role in leishmaniasis; on one hand they serve as host cells, propagating the infection and indirectly hindering antigen presentation (Peters et al., 2008; Hurrell et al., 2015), on the other hand they have been shown to contribute to parasite killing by releasing neutrophil extracellular traps (Mougneau et al., 2011).

## Host Immunology

A protective immune response against leishmaniasis is characterized by a CD4+ Th1-polarized immune response (Centers for Disease Control and Prevention, 2013). Upon interaction with parasites, antigen presenting cells (APCs) activate T-cells by direct contact as well as by the release of disease-protective cytokines such as interleukin-12 (IL-12). These cytokines prime naïve T-helper cells to differentiate into Th1 cells, the main producers of interferon-γ (IFN-γ) (Naman et al., 2018). Natural killer (NK) cells is an another important source of IFN-y that contributes to Th1 cell differentiation and ultimately to disease resolution (Mougneau et al., 2011). IFN- γ stimulates phagocytes to produce reactive oxygen- and nitrogen-species resulting in parasite killing (Pace, 2014). IFN-γ, IL-12 and TNF-α are crucial mediators of protection against various forms of *Leishmania* infection (Naman et al., 2018; World Health Organization, 2018). While a polarized Th1 response is associated with resolution of CL, susceptibility to CL is associated with an induction of a Th2 immune response. A Th2-polarized immune response is characterized by the production of interleukin-4 (IL-4), IL-13 and interleukin-10 (IL-10). These cytokines, along with TGF-β suppress protective immune response and promote parasite and exacerbation of disease (Odiit et al., 1997). In contrast to CL, the resolution of VL requires the production of interleukin-4 (IL-4) and interleukin-13 (IL-13) as well as IL-4 α signaling pathway, which induces mature granuloma formation and promotes parasite clearance (Satoskar et al., 1995; Alexander et al., 2000; Stager et al., 2003; McFarlane et al., 2011).

Several drugs are currently used to treat leishmaniasis; but there is no vaccine available for disease prevention. The standard in treatment of leishmaniasis in most endemic countries involves use of pentavalent antimonials such as sodium stibogluconate (SSG). Other drugs, including liposomal amphotericin B, azoles, imiquimod, miltefosine, paromomycin and pentamidine, have also been used with variable success. Unfortunately, the current treatments have several drawbacks, including poor patient compliance due to prolonged treatment duration, high toxicity and emergence of drug resistant parasites (Eugene, 1987; Bray et al., 2003; Torrado et al., 2008; Jhingran et al., 2009; Olliaro and Sundar, 2009; Tiuman et al., 2011; McGwire and Satoskar, 2014; Lamotte et al., 2017; Ponte-Sucre et al., 2017). In this section, we will discuss novel host-targeted drugs to treat leishmaniasis.

## Host-Targeted Therapeutics and Approaches for Treatment of Leishmaniasis

Imatinib is an inhibitor of Abl/Arg kinase family of tyrosine kinases, which can directly remodel the actin-based cytoskeleton to mediate phagocytosis (Greuber and Pendergast, 2012; Zhang and Kima, 2016). The Abl/Arg kinases have been previously shown to play a role in phagocytosis of *Leishmania amazonensis* promastigotes by macrophages (Wetzel et al., 2012). Although treatment with imatinib did not significantly alter cytokine production, reduced the uptake of both opsonized and non-opsonized parasites and led to reduced lesion severity in mice (Wetzel et al., 2012).

Phosphoinositide 3-kinase γ (PI3Kγ) is part of a family of enzymes with the function of phosphorylating lipids containing phosphotidylinositol. PI3Kγ is expressed in leukocytes and mediates cell migration by initiating actin cytoskeletal reorganization. Because cytoskeletal rearrangement is also critical to phagocytosis, blocking or deleting PI3Kγ results in a significant impairment of parasite entry into phagocytic host cells *in vitro* and *vivo*. This decreased phagocytosis, along with an impaired recruitment of cells at the infection site, conferred increased resistance against *L. mexicana* in C57BL/6 mice. Furthermore, AS-605240, an isoform-selective PI3kγ inhibitor, was therapeutically as effective as aforementioned standard anti-leishmanial drug SSG in treating *L. mexicana* infection (Cummings et al., 2012). A recent study used AS101 (ammonium trichloro [1,2-ethanediolato-O,O’]-tellurate), a tellurium-based immunomodulator for the treatment of *L. donovani* infection. Along with a direct effect on promastigotes, AS101 was also shown to reverse T-cell anergy, promote NO and antibody production, and more importantly inhibit the STAT3/IL-10 pathway by blocking the PI3k/Atk signaling in infected macrophages. This could further promote MAPK and NF-κB activity (Vishwakarma et al., 2018). A recent study by Khadem et al. (2017), showed that administration of PI3K p110δ inhibitors CAL-101 and IC87114 resulted in a decrease in parasitic burden in both a CL and VL murine model. This result was accompanied by increased cytokine production in the spleen, livers and footpads of infected mice. The authors suggest the use of these inhibitors along with amphotericin B for even better outcomes (Khadem et al., 2017).

Ibrutinib is a small inhibitor currently used for the treatment of chronic lymphocytic leukemia and other B-cell malignancies due to its action as an irreversible inhibitor of Bruton’s tyrosine kinase (BTK) found on B-cells (Pan et al., 2007; Harrison, 2012). Because of its homology with BTK, IL-2 inducible kinase (ITK) found on both Th1 and Th2 cells is also inhibited by ibrutinib (Dubovsky et al., 2013). Previous studies have identified ibrutinib as a clinically relevant drug not only against cancer, but also for the treatment of infectious disease using the cutaneous model of leishmaniasis caused by *L. major*. This beneficial effect was due to a Th1 polarized response characterized by the production of disease protective cytokines such as IFN-γ (Dubovsky et al., 2013).

Berberine chloride is a quaternary isoquinoline that acts via phosphorylation of protein p38 in the MAP kinase pathway. This compound upregulates NO production and IL-12 expression, both disease protective, while downregulating expression of disease exacerbating IL-10 in macrophages infected with *L. donovani* (Saha et al., 2011). Berberine also alters AMP-activated protein kinase (AMPK) signaling, leading to increased activation of macrophage inflammasomes (Casey et al., 2015; Li et al., 2017).

Statins are HMG-CoA reductase inhibitors commonly orally administered to decrease low density lipoprotein (LDL) levels in hyperlipidemic individuals by preventing the synthesis of cholesterol in the liver (Sirtori, 2014). During *L. donovani* infection, this statin-dependent reduction in cholesterol levels resulted in decreased attachment of infectious promastigotes to macrophages, which is critical for parasite invasion. Consequently, macrophages treated with lovastatin had fewer intracellular amastigotes (Kumar et al., 2016). Furthermore, topical application of simvastatin to ear and footpad lesions of *L. major*- infected BALB/c and C57BL/6 mice reduced lesion size as well as parasitic burdens in draining lymph nodes (Parihar et al., 2016).

Naloxonazine is an opioid-receptor antagonist that up-regulates expression of the vacuolar ATPase (vATPase) proton pump and actin related genes, mediating the formation and maturation of phagolysosomes. The vATPase proton pump has been shown to be critical in acidification of parasitophorous vacuoles within phagocytes. The general understanding is that promastigotes are more sensitive to acidic pH than amastigotes. This observation suggests that acidification of vacuole before the transformation of promastigotes into amastigotes can potentially lead to increased parasite killing (De Muylder et al., 2016).

Pentalinonsterol (cholest-4,20,24-trien-3-one) is a natural product isolated from the roots of *Pentalinon andrieuxii*, a native plant of the Yucatan peninsula. Recent studies have shown that Pentalinonsterol can stimulate macrophages by activating the NF-kB pathway. This activation resulted in an upregulation of NO, critical for parasitic killing, as well as pro-inflammatory cytokines TNF-α and IL-12 in macrophages and bone marrow derived macrophages (BMDMs) *in vitro*. Pentalinonsterol treatment also increased antigen presentation and expression of costimulatory molecules, which ultimately resulted in augmentation of both the responses *in vivo*. Because of its immunomodulatory properties, pentalinonsterol has been proposed as a potential adjuvant in vaccination against infectious diseases (Oghumu et al., 2017).

Oleuropein is a glycosylated seco-iridoid that can be cheaply derived from numerous plants, in particular the olive tree, *Olea europaea L.* (Oleaceae). This natural bioactive compound was shown to promote Th1 type immune responses and increase the oxidative stress within the host, both important for protection against *Leishmania* infection. Balb/c mice infected with *L. donovani* and subsequently treated with oleuropein showed a Th1 polarization characterized by expression of genes like TGF-β1 and IFN-γ as well as transcription factors like GATA3 (Kyriazis et al., 2016). This immunomodulatory effect was believed to be due to the inhibition of IL-1β which promotes disease progression and non-healing phenotypes in *Leishmania major* infections (Voronov et al., 2010; Charmoy et al., 2016). In addition to these properties, oleuropein treatment increased the production of ROS in both *in vitro* and *in vivo* models of *L. donovani* infection (Kyriazis et al., 2016). Oleuropein has also been shown to inhibit extracellular signal related kinase (ERK1/2) (Abe et al., 2011). This is relevant because activation of ERK1/2 enhances expression of IL-10 and reduction of IL-12 which resulted in decreased p38 MAPK activation and increased parasite survival (Feng et al., 1999; Mathur et al., 2004). Interestingly, oleuropein has stimulatory effects on the AMPK pathway similar to berberine chloride (Andreadou et al., 2014). Because of its immunomodulatory actions and low toxicity, oleuropein could be employed to complement other treatments.

Mahanine is a carbazole alkaloid isolated from a medicinal plant native to the Indian subcontinent. *In vivo* studies have shown that mahanine induces apoptosis of both antimony sensitive and resistant *L. donovani* (Roy et al., 2017). Mahanine augments NO and ROS generation, thereby causing parasitic apoptosis due to oxidative stress. Along with its effect on NO and ROS, mahanine affects Th1 cytokines by acting on the STAT pathway. First employed in the treatment of various types of cancer, mahanine has been effectively repurposed against VL (Roy et al., 2017). Mahanine inhibits JAK1 and Src which subsequently promotes the degradation of STAT3, an important transcription factor in macrophages that causes upregulation of IL-10 expression and suppression of Th1 responses (Biswas et al., 2011; Lee et al., 2011; Das et al., 2014). While mahanine possesses its own unique immunomodulatory effects, several other natural compounds stimulate ROS and NO production *in vitro* including but not limited to, lupeol from *Sterculia villosa*, dehydroabietic acid from *Pinus elliottii*, and oil extracts from *Nectandra* species (da Costa-Silva et al., 2015; Bosquiroli et al., 2017; Das et al., 2017; Goncalves et al., 2018). For example, *Punica granatum*, commonly known as pomegranate, has been shown to have antiparasitic and antioxidant properties (Kaur et al., 2006). A recent study has demonstrated that oral treatment with *P. granatum* juice significantly reduced the lesion sizes of mice infected with *L. major*, compared to untreated mice. The anti-leishmanial activity is attributed to the presence of flavonoid and phenolic compounds including ellagitannins and luteolin. Luteolin in particular inhibits the extracellular promastigote growth (Mittra et al., 2000). In contrast, ellagitannins enhance non-specific immunity via macrophage activation and by inducing the production of NO, IFN-γ and TNF-α, which increase the oxidative stress on the parasites. The study showed that *P. granatum* juice has the potential to be an effective, safe and easily administrable treatment against CL (Alkathiri et al., 2017).

Fucoidan is a multi-sulfated polysaccharide isolated from the sporophylls of the brown algae *Undaria pinnatifida*. Fucoidan enhances dendritic cell (DC) maturation through increased expression of MHC-II and co-stimulatory molecules such as CD80/86 and CD40 (Jin et al., 2014). Fucoidan treatment of DCs induced secretion of IL-6, IL-12, TNF-α and IFN-γ with notable increases in NO production and decreases in production of the anti-inflammatory cytokines IL-10 and TGF-β (Yang et al., 2008; Kar et al., 2011; Jin et al., 2014). Over 93% inhibition of *L. donovani* amastigote replication was achieved *in vitro* and parasites were cleared from both the liver and spleen in 6 weeks *in vivo*; the reduction of parasite burden was observed in both antimony susceptible and resistant *L. donovani* strains (Kar et al., 2011). Fucoidan activates p38 and ERK1/2 associated NF-κB signaling in *L. donovani*-infected macrophages (Sharma et al., 2014).

Artemisinin is a natural compound found in *Artemisia annua*, a traditional Chinese medicinal herb that is the mainstay for treatment of malaria. Treatment with Artemisinin-loaded poly lactic co-glycolic acid (ALPLGA) nanoparticles was shown to increase levels of IL-2 and IFN-γ and decrease levels of IL-4 and IL-10 in BALB/c mice infected with *L. donovani* (Want et al., 2015). Artemisinin treated macrophages secreted increased levels of IL-12 *in vitro* through inhibition of JNK signaling (Cho et al., 2012). Treatment of *L. donovani-*infected BALB/c mice with artemisinin-conjugated nanoparticles and liposomal preparations resulted in approximately 80% reduction of spleen and liver parasite burdens after one administration (Want et al., 2015; Want et al., 2017). Furthermore, treatment of mice with the semi-synthetic derivative, dihydroartemisinin, has been shown to decrease the number of T-regulatory cells, which are important mediators of *Leishmania* pathogenesis present in the spleen, in addition to having Th1 polarizing effects (Belkaid, 2003; Noori and Hassan, 2011). Oral administration of *Artemisia annua* powder in gelatin capsules drastically reduced lesion size and improved appearance compared to no treatment in hamsters infected with *L. panamensis*; 5 out 6 hamsters treated with *A. annua* capsules daily for 30 days were completely cured and 2 clinical patients taking *A. annua* capsules were cleared of infection within 45 days without adverse reactions or reoccurrence 24 months after completion of therapy (Mesa et al., 2017).

Eugenol is a component of *Syzygium aromaticum*, a species of clove native of Australia and tropical regions of Central and South America. *S. aromaticum* has anti-bacterial, anti-trypanosomal and anti-malarial activity. These properties are due to the immunomodulatory effects of this compound on both humoral and cell-mediated immune responses. Treatment of *L. donovani*-infected BALB/c mice with eugenol increased Th1 polarization, characterized by high levels of IFN-γ and IL-2, with a concomitant decrease in the Th2 immune response (Charan Raja et al., 2017). This treatment also induced nitric oxide production in infected macrophages and proliferation of both CD4+ and CD8+ T-cells. Overall, eugenol treatment resulted in decreased parasitic burdens in the spleen and livers of *L. donovani*-infected mice most likely due to both leishmanicidal and immunomodulatory properties (Islamuddin et al., 2016; Charan Raja et al., 2017).

Brazilian propolis has recently been investigated for the treatment of American tegumentary leishmaniasis. Propolis is a bee product composed mainly of di-triterpens, phenolic compounds and essential oils. This natural product has previously been shown to have anti-inflammatory, anti-oxidant and immunomodulatory properties (Miranda et al., 2015). In a study from 2015, it was shown that treatment with NO in combination with Brazilian propolis mediated a decrease in number of parasitized cells, leading to reduced inflammation and tissue damage in a *L. amazonensis* murine model (Miranda et al., 2015). More recently, the immunomodulatory properties of Brazilian propolis was investigated in human-derived peripheral blood mononuclear cells (PBMC) isolated from *L. braziliensis* infected patients. This study found that in both healthy and infected donors, propolis was able to increase the levels of IL-4 and IL-17 while decreasing IL-10, showing an overall decrease in inflammation which could promote the control of the parasites (Dos Santos Thomazelli et al., 2017).

Phospholipase A_2_ (PLA_2_) are a family of enzymes with the function of reacting with various phospholipids to produce lysophospholipids, a class of lipid mediators, as well as arachidonic acid, a precursor of eicosanoids (Murakami and Kudo, 2002; Moreira et al., 2014). It has been previously shown that PLA_2_ activates NF-kB in isolated peritoneal macrophages (Moreira et al., 2014). Additionally, treatment with liposome encapsulated PLA_2_ isolated from the venom of *Bothrops jararacussu*, resulted in a significant rise in TNF-α and NO production in the cutaneous lesions as well as lymph nodes of *L. amazonensis*-infected BALB/c mice (de Barros et al., 2018).

Leptin is an adipocyte hormone that plays a role in thymic homeostasis and has been shown to mediate a pro-inflammatory response in animal models. In particular, leptin induces Th1 immune responses while suppressing Th2 responses (Maurya et al., 2016). Leptin induced Th1 polarization, characterized by IFN-γ, IL-12 and IL-1β, resulted in decreased parasitic burdens of C57BL/6 mice infected with *L. donovani* (Dayakar et al., 2016; Maurya et al., 2016). Leptin treatment also mediated NO production in antigen-presenting cells (Maurya et al., 2016). In particular, leptin activates macrophages by promoting the phosphorylation of ERK1/2 and Akt, which is usually inhibited during VL infection (Dayakar et al., 2016).

**FIGURE 1:**
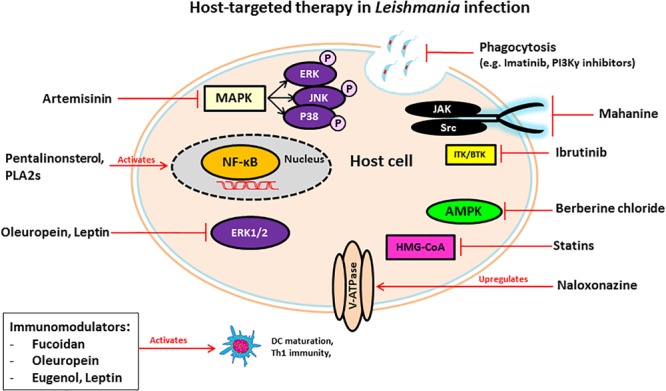
Schematic diagram of mechanisms of host-targeting against Leishmania infection.

MicroRNAs (miRNAs) are short, single-stranded non-coding RNAs that bind to gene transcripts to regulate protein translation (Bartel, 2009; Geraci et al., 2015). Because of their role in mediating post-transcriptional repression, miRNAs are linked to the regulation of host processes involved in the development and activity of innate and adaptive immune responses (Geraci et al., 2015; Drury et al., 2017). The immunomodulatory functions of miRNAs make them promising host targets for developing new therapeutics for infectious disease (Drury et al., 2017). miRNAs have been implicated in visceral leishmaniasis infection in macrophages and dendritic cells *in vitro* (Geraci et al., 2015). Additionally, certain miRNAs have been involved in T-reg specialization and stability in an *L. major* model, and in autophagy in both *L. major* and *L. donovani* models (Kelada et al., 2013; Frank et al., 2015; Singh et al., 2016). *L. donovani* infection was found to increase the stability of microRNA ribonucleoprotein (miRNP) in infected macrophages. miRNP works to restrict the production of pro-inflammatory cytokines, detrimental for parasite survival within the host cell (Chakrabarty and Bhattacharyya, 2017). For these reasons, miRNAs can serve as drug targets to manipulate the host immune response to pathogens. We summarized the compounds in this section and their targets in Table 1 and their detailed mechanism of action in Figure 1.

**Table 1:** Host-targeted therapeutics for leishmaniasis.

Host-targeted Drug	Classification	Mode of action	Reference
Imatinib	Abl/Arg kinase inhibitor	Plays a role in phagocytosis	Wetzel et al., 2012
Phosphoinositide 3-kinase γ (PI3Kγ) inhibitor	PI3Kγ inhibitor	Inhibits actin cytoskeletal reorganization	Cummings et al., 2012
Ibrutinib	Bruton tyrosine kinase (BTK), and IL-2–inducible kinase (ITK) inhibitor	Results in a Th1 polarized immune response	Dubovsky et al., 2013
Berberine chloride	Hydrochloride salt	Up-regulates nitric oxide and IL-12 production while downregulating IL-10 production	Casey et al., 2015; Li et al., 2017
Statins	HMG CoA reductase inhibitors	Leads to reduced numbers of promastigotes attached to host cells	Kumar et al., 2016; Parihar et al., 2016
Naloxonazine	Opioid receptor antagonist	Up-regulates the expression of the vacuolar ATPase (vATPase) proton pump to acidify the vacuole	De Muylder et al., 2016
Pentalinosterol	Natural compound – cholest-4,20,24-trien-3-one	Activates macrophages to up-regulate pro-inflammatory cytokine and nitric oxide production	Oghumu et al., 2017
Oleuropein	Natural compound – glycosylated seco-iridoid	Promotes a Th1 type immune response and increases oxidative stress	Voronov et al., 2010; Abe et al., 2011; Charmoy et al., 2016; Kyriazis et al., 2016
Mahanine	Natural compound – carbazole alkaloid	Modulates Th1 cytokines and promotes oxidative stress	Biswas et al., 2011; Lee et al., 2011; Das et al., 2014, 2017; da Costa-Silva et al., 2015; Bosquiroli et al., 2017; Roy et al., 2017; Goncalves et al., 2018
Punica granatum (pomegranate)	Natural compound – flavonoids and phenolic compounds	Activates macrophages to increase oxidative stress by inducing Th1 cytokines	Kaur et al., 2006; Mittra et al., 2000; Alkathiri et al., 2017
Fucoidan	Natural compound – multi-sulfated polysaccharide	Enhances DCs maturation and stimulates production of pro-inflammatory cytokines while down-regulating anti-inflammatory cytokines	Yang et al., 2008; Kar et al., 2011; Jin et al., 2014; Sharma et al., 2014
Artemisinin	Natural compound – sesquiterpene lactone containing a peroxide bridge	Increases levels of IL-2 and IFN-γ and decreases levels of IL-4 and IL-10	Belkaid, 2003; Noori and Hassan, 2011; Cho et al., 2012; Want et al., 2015, 2017
Eugenol	Natural compound – phenylpropene	Mediates Th1 polarized response	Islamuddin et al., 2016; Charan Raja et al., 2017
Propolis	Natural compound – mainly flavonoids, aromatic acids and benzopyranes	Decreases inflammation	Miranda et al., 2015; Dos Santos Thomazelli et al., 2017
Phospholipase A_2_	Enzyme that produces lipid mediators	Promotes activation of NF-kB in macrophages and results in increased nitric oxide and TNF-α production	Murakami and Kudo, 2002; Moreira et al., 2014; de Barros et al., 2018
Leptin	Adipocyte hormone	Induces a Th1 polarized response and augments nitric oxide production	Dayakar et al., 2016; Maurya et al., 2016
Micro RNA targets (miRNA)	mRNA	Act as immunomodulators	Bartel, 2009; Kelada et al., 2013; Frank et al., 2015; Geraci et al., 2015

## Chagas Disease (American Trypanosomiasis)

*Trypanosoma cruzi* is the causative agent of American trypanosomiasis (Chagas disease). This infection has a prevalence of nearly 6-8 million people worldwide (Aiga et al., 2009) and causes about 12,000 deaths annually. Although Chagas disease is endemic in certain areas in Central and South America, it is found worldwide through the migration of chronically infected individuals. Additionally, there is a low incidence of parasite-infected triatomine bugs in the lower 20 states in the United States which occasionally infect humans (Bern et al., 2011; Klotz et al., 2014).

*Trypanosoma cruzi* infection is most often initiated during blood feeding of parasite-infected triatomine bugs which defecate near the feeding site. Metacyclic trypomastigotes present in the insect feces enter the wound and establish infection. Metacyclic trypomastigotes are able to invade any nucleated host cells before transforming into intracellular amastigotes and replicating (Rassi et al., 2010). Amastigotes eventually differentiate into blood-stage trypomastigotes and exit the host cell. Trypomastigotes and intracellular amastigote-laden host cells disseminate within the host and can infect multiple organ systems. Key end organ tropisms for parasites are cardiac and gastrointestinal smooth-muscle. The life cycle is complete when uninfected triatomine bugs feed on infected mammalian hosts and ingest parasites, which grow and differentiate in the insect gastrointestinal tract and eventually migrate to the hindgut. The majority of Chagas infections occur through insect vector-borne transmission; however, the disease can also be transmitted trans-placentally, through blood and tissue transplantation, through the consumption of parasite-laden meat or contaminated freshly squeezed fruit juice and through accidental laboratory exposure (Tyler and Engman, 2001; de Souza et al., 2010; Bern et al., 2011).

The disease has two distinct stages, acute and chronic. The acute stage lasts for 4–8 weeks and generally goes unnoticed or it presents with mild symptoms such as fever, headache, fatigue, and/or rash. Once the infection is established, most patients undergo chronic infection. Among chronically infected patients, 60–80% of individuals will develop an indeterminate chronic stage without showing any symptoms. The remaining 20–40% will eventually develop significant cardiac or gastrointestinal symptoms including arrhythmias and cardiomyopathy resulting in congestive heart failure, and gastrointestinal tract dysmotility syndromes leading to symptoms associated with achalasia, mega-colon and mega-esophagus (Sanchez-Guillen Mdel et al., 2006; Bern et al., 2011).

Only two drugs available for treatment of Chagas disease are nifurtimox and benznidazole (Bern et al., 2007). These drugs are not completely effective and their use is difficult due to toxic side effects (Castro et al., 2006). Thus, there is an urgent need to develop new drugs and vaccines for the treatment and control of Chagas disease. Host-targeted therapy could provide an alternative approach to treat Chagas disease in the future. Here, we focus on some of the possible host molecular targets that can be exploited to treat Chagas disease.

## Host Immunology

*Trypanosoma cruzi* invasion of host cells, intracellular growth and parasite release eventually elicits a rise in parasitemia, which in turn induces pro-inflammatory responses by macrophages and natural killer cells and results in strong CD8+ T cell immune responses (Tarleton, 2015). However, these immune responses can only control the infection partially, as a low level of infection persists for the entire life of the host. During the acute phase of infection, the Th1 response is involved in protection (Tarleton et al., 2000). Driven by interleukin 2 (IL-2) and interferon gamma (IFN-γ) produced by Th1 cells, this type of immune response is important in resistance against *T. cruzi* infection, whereas a Th2 polarized response mediates parasite persistence. IFN-γ-mediated protection is regulated by the transcription factor STAT-1 (signal transducer and activator of transcription 1), and the lack of STAT-1 has been shown to increase susceptibility to *T. cruzi* infection in mice (Stahl et al., 2014; Kulkarni et al., 2015).

Glycosylphosphatidylinositol (GPI)-anchored mucin-like glycoprotein from *T. cruzi* plays a crucial role in macrophage activation, mediating stimulation of pro-inflammatory cytokines such as TNF-α, IL-12, and also inducing NO synthesis in innate immune cells (Camargo et al., 1997). A recent study shows that Th17, a subset of CD4+ T cells, provides a stronger protective response than Th1 cells against *T. cruzi* infection. Th17-dependent protection is due to the phagocytic respiratory burst as well as the activation of CD8+ T cells (Cai et al., 2016). Thus, the combined effect of cell-mediated immune unbalanced response associated continuous subpatent parasite antigens may play a significant role in the development of the pathogenesis of Chagas disease. Therefore, host-directed drugs modulating host immune response could be a viable therapeutics for managing Chagas disease.

## Host-Targeted Therapeutics and Approaches for Treatment of Chagas Disease

While host-targeted therapy in *T. cruzi* infection has not been well studied, there are several studies that show different host molecules are critical for the establishment of *T. cruzi* infection, and the inhibition of these molecules may reduce or ablate infection.

G-protein coupled receptors are a family of receptors that utilize G-proteins to transduce signals into the cell and control diverse functions, including regulation of gene transcription, cellular motility, and metabolic enzymes. *T. cruzi* trypomastigotes invade host cells through association with various GPCRs including platelet-activating factor receptor (Kawano et al., 2011), bradykinin receptor B1 and B2 (Scharfstein et al., 2000; Todorov et al., 2003) and muscarinergic 2 receptor (Wallukat et al., 2010). Several inhibitors of GPCRs have been shown to prevent *T. cruzi* entry and infection and mediate protection against Chagas disease. Cannabinoids, a family of potent immunosuppressive agents, inhibit G-protein signaling and invasion of cardiac myoblasts by *T. cruzi* in mice (Croxford et al., 2005). It is known that parasite-derived thromboxane A2 (TXA2) is important for disease progression in Chagas disease (Villalta et al., 2009). Intracellular amastigotes release TXA2 and initiate signaling after binding with TXA2 receptor (TP). Binding of TXA2 and TP activates a variety of cell types including dendritic cells, monocytes, platelets, cardiac myocytes and endothelial cells, resulting in apoptosis of cells, vasoconstriction, dilated cardiomyopathy, enhanced platelet adherence and aggregation (Ashton et al., 2007). The TXA2 receptor antagonist SQ29548 has been shown to inhibit *T. cruzi* infection mediated through TP (Ashton et al., 2007; Silva et al., 2016).

Carvedilol is a non-selective β-adrenergic receptor blocker used to manage congestive heart failure. It has been shown to improve Chagas cardiomyopathy in combination with renin-angiotensin inhibitors (Botoni et al., 2007).

SB-431542 compound is an inhibitor of the TGF-β type I receptor kinase. It has been shown that host TGF-β is increased during *T. cruzi* infection (Silva et al., 1991) and is taken up by amastigotes to modulate the life cycle of *T. cruzi* (Araujo-Jorge et al., 2002; Waghabi et al., 2005). Recently, it has been reported that elevated TGF-β causes the heart fibrosis and severe cardiomyopathy in Chagas disease. These findings suggest that the treatment of cardiomyocytes with SB-431542, can inhibit the effect of TGF-β-mediated amastigote proliferation and cardiac myopathy in Chagas disease. Experimental evidence suggests that treatment with this drug lowers the penetration of trypomastigotes into cardiomyocytes, decreases intracellular amastigote multiplication and trypomastigote release from the cells, reducing the severity of infection and mortality of mice (Waghabi et al., 2007, 2009).

Terpenoides possess anti-trypanosomal activity. Treatment with terpenoid compounds, such as cumanin and psilostachyin, reduces parasitemia and mortality of parasite-infected mice and intracellular amastigote replication in Vero cells (Sulsen et al., 2013). Terpenoides are a widespread group of natural products and potent inhibitors of NF-κB signaling which mediates TNF-α-induced cell death. During early stages of infection, *T. cruzi* invade hepatocytes, macrophages, and Kupffer cells and increase TNF-α production that causes apoptotic cell death of infected hepatocytes (Ronco et al., 2010). Furthermore, infection of *T. cruzi* leads to activation of host cell NF-κB signaling that protects infected cells from undergoing apoptosis (Petersen et al., 2006). As terpenoides are such potent inhibitors of NF-κB, treatment might result in robust apoptotic cell death of infected cells that release intracellular parasites outside of the cells. Although the exact mechanism of the trypanocidal effect of terpenoides is still unknown, they may target released parasites from apoptotic host cells directly and engage in anti-trypanosomal activity. In contrast, da Silva et al. (2013) showed that the sesquiterpene lactones-psilostachyin and cynaropicrin did not have efficacy in the mouse model of acute *T. cruzi* infection when comparing benznidazole (da Silva et al., 2013).

Immuno-modulators are compounds that modify immune responses. Extracts from *Lycopodium clavatum*, a spore bearing vascular plant, act as an immuno-modulator to induce Th1 immune responses. *T. cruzi* infected rats treated with *Lycopodium clavatum* extract have reduced progression of GI tract Chagas disease (Brustolin Aleixo et al., 2017). A recent study by Otta et al. (2018) showed that K777 the extract lead compound induces prominent proinflammatory responses modulation by interleukin -10-positive CD4^+^/CD8^+^ T cells and this contributed to the protection against the Chagas disease. In addition, diet supplementation with fish oil led to increase the resistance to *T. cruzi* infection through modulating various immunological factors (Lovo-Martins et al., 2017).

**FIGURE 2:**
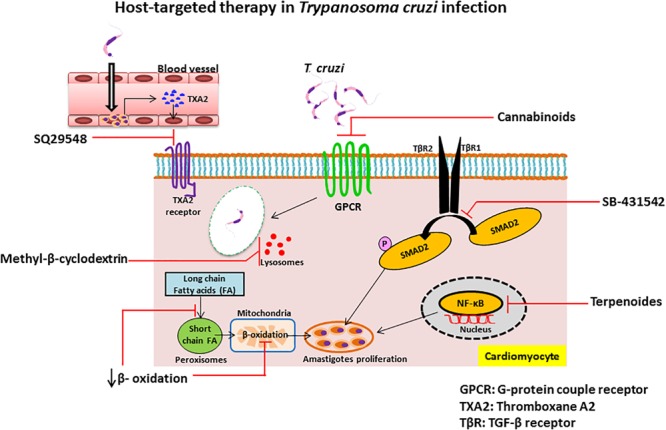
Schematic diagram of mechanisms of host-targeting against *Trypanosoma cruzi* infection.

Inhibition of β-oxidation: The replication of intracellular amastigotes is largely supported by parasite scavenging of host metabolic network, including host cell fatty acid metabolism in cardiac and smooth muscle (Combs et al., 2005). It has been shown that long chain fatty acid oxidation is the key source of nutrients for intracellular amastigotes. Long chain fatty acids are oxidized in the peroxisome to produce short chain fatty acids that are transported to the mitochondria by acyl-CoA dehydrogenase for β-oxidation. Recent study of siRNA screen shows that the enzymes of β-oxidation are essential for growth of amastigotes inside the host cells and alteration of fatty acid metabolism and β-oxidation inhibits the intracellular growth of amastigotes (Caradonna et al., 2013). Although several β-oxidation inhibitors have been identified as, for example etomoxir (Paumen et al., 1997), mildronate (Liepinsh et al., 2006), trimetazidine, and ranolazine (Sabbah and Stanley, 2002), more studies are needed to find which can treat Chagas disease. We summarized the compounds in this section and their targets in Table 2 and their detailed mechanism of action in Figure 2.

**Table 2:** Host-targeted therapeutics for Chagas disease.

Host-targeted Drug	Classification	Mode of action	Reference
Cannabinoids, SQ29548	inhibitors	G protein couple receptor	Scharfstein et al., 2000; Todorov et al., 2003; Croxford et al., 2005;Ashton et al., 2007; Wallukat et al., 2010; Kawano et al., 2011
Carvedilol	β-adrenergic receptor blocker	β-adrenergic receptor	Botoni et al., 2007
SB-431542 compound	inhibitor	TGF-β type I receptor kinase	Silva et al., 1991; Araujo-Jorge et al., 2002; Waghabi et al., 2005; Waghabi et al., 2007
Terpenoides	Inhibitors	NF-κB signaling	Petersen et al., 2006; Ronco et al., 2010; da Silva et al., 2013; Sulsen et al., 2013
*Lycopodium clavatum*	immunomodulator	Induces Th1 immune responses	Brustolin Aleixo et al., 2017; Lovo-Martins et al., 2017; Otta et al., 2018

## Human African Trypanosomiasis (Sleeping Sickness)

Human African trypanosomiasis (HAT) is caused by two subspecies of *Trypanosoma brucei, T. b. gambiense*, and *T. b. rhodesiense*. *T. b. gambiense* is the causative agent of a chronic form of the disease often referred to as West African trypanosomiasis, which is prevalent in Western and Central Africa and affects 24 countries. *T. b. rhodesiense* causes a more acute form of the disease found in Eastern and Southern Africa affecting 13 countries, and is often referred to as East African trypanosomiasis (Franco et al., 2014).

Of the two subspecies, *T. b. gambiense* is the more prevalent threat, as humans are considered the primary reservoir for the parasite. *T. b. rhodesiense* primarily infects animal reservoirs, and humans are incidentally infected (Franco et al., 2014). According to the World Health Organization (WHO), the majority of approximately 2,000 new cases of Western African trypanosomiasis reported in 2016 were in the Congo, while only about 50 new cases were found in Eastern Africa (World Health Organization, 2017). Currently, there are estimated to be fewer than 20,000 active cases of sleeping sickness, with 65 million at risk. The majority of new cases occur in the Democratic Republic of Congo, contributing 84% of new cases in 2015 (Franco et al., 2014). HAT is slated for elimination as a public health threat in 2020, with a downward trend in new cases and a drop of yearly DALY from 2,734 DALY in the year 2000 to a DALY of 372 in year 2015 (World Health Organization, 2013, 2016, 2017).

Both subspecies of *T. brucei* are transmitted by Tsetse flies harboring metacyclic trypomastigotes, while feeding on mammalian hosts. Once inside the host, trypomastigotes migrate to the blood stream and lymphatics disseminate throughout the host, at which point they begin to multiply through binary fission. (Centers for Disease Control and Prevention, 2016). In acute human trypanosomiasis, parasites disseminate throughout the lymphatics and blood stream of the host. Parasites eventually breach the blood-brain barrier, leading to infection of the central nervous system, and eventual death in essentially 100% of untreated patients (Brun et al., 2010).

## Host Immunology

Upon entering the human host, metacyclic *T. brucei* immediately encounter the host’s innate immune defense mechanisms. These parasites, however, are covered in a thick, highly dense coat of variable surface glycoproteins (VSGs) which protect parasites from the mounting host humoral response, as well as from host complement-mediated lysis (Horn, 2014). Parasites undergo switching of antigenically different VSGs in order to circumvent antibody-mediated killing. This switching of VSGs results in undulating waves of parasitemia in which parasites possessing older VSG coats are subject to immune clearance and clones expressing neo-VSG escape immune surveillance and replicate. This mechanism allows the trypanosomes to replicate and survive at sub-lethal levels of infection.

Human susceptibility, disease progression and outcome of HAT are linked to differences in individual genetic predisposition for varying cytokine levels and T cell differentiation. Once infected, certain individuals can control infection and become asymptomatic carriers without any specific intervention. These asymptomatic people have increased quantities of IL-6, IL-8 and TNFα, as well as decreased levels of IL-12, indicating that the ability to control infection relies upon a controlled Th1 response. However, those individuals showing high levels of IL-6, IL-8, TNFα, and IL-10 are at risk of developing disease once infected (Courtin et al., 2006; Kato et al., 2015, 2016). In contrast, the individuals who show more susceptibility to uncontrolled infection produce higher amounts of IL-2 and IL-4. Control of late infection once it has been established, relies upon the immune system being able to effectively switch from a Th1 to a Th2 immune response (Ilboudo et al., 2014). While the Th1 response is more beneficial during the initial stages of infection, more anti-inflammatory Th2 responses are implicated in trypano-tolerance once an infection has been established. IFN-γ expression is responsible for early resistance and control against initial infection (Hertz et al., 1998). While IFN-γ is beneficial to the host during initial stages of infection, the lack of effective switching to a more polarized Th2 response during the late stages of infection may lead to hyper-inflammation in the CNS, ultimately overwhelming the host (Shi et al., 2003).

In addition to the Th1 and Th2 response, high concentrations of VSG released by the parasites in the blood stream play an important role in inducing host immune response. Glycosylphosphatidyl-inositol (GPI)-phospholipase C induced release of GPI-linked VSGs exposes macrophages to previously masked regions of the GPI-tails of VSG. GPI-recognition by macrophages induces MyD88 dependent activation of the NF-κB cascade resulting in a massive release of TNF-α, IL-1, and IL-12 (Leppert et al., 2007; Cheung et al., 2016; Stijlemans et al., 2016). This release of pro-inflammatory cytokines alongside parasite components results in the induction of classically activated macrophages. These macrophages release ROS and NO which can be detrimental to parasites, however, they are also damaging to the host, causing physiological and cellular destruction (Stijlemans et al., 2016).

## Host-Targeted Therapeutics and Approaches for Treatment of Hat

Current treatment approaches of HAT consist of 5 drugs, each one specific for different stages of the infection. Pentamidine and suramin are used to treat the first stage of the disease, whereas melarsoprol and a combination of nifurtimox-eflornithine are employed to treat the second stage of the disease. All these drugs have significant potential side effects. Pentamidine treatment is fairly ineffective in combating the second stage of infection by *T. b. gambiense* and both stages of *T. b. rhodesiense*, suramin treatment is only effective against the first stage of *T. b. rhodesiense* infection (Buscher et al., 2017).

Melarsoprol is employed to treat the late-stage of infections caused by both *T. b. gambiense* and *T. b. rhodesiense*, and is the only drug used for late-stage infection caused by *T. b. rhodesiense*. However, significant toxicity, the mode of administration and the lack of its availability in endemic areas, hinder widespread usage of this drug. Although nifurtimox and eflornithine are individually effective against *T. b. gambiense* infection, combination therapy with these drugs has better efficacy and diminished side effects (Barrett et al., 2007; Priotto et al., 2009; Babokhov et al., 2013).

There are currently three drugs under development for HAT, as well as a few host target molecules which have shown promising results in the reduction of parasitemia.

Diamidine derivatives, including pafuramidine (DB289), which is administered orally, is well tolerated and mediates parasite clearance in late-stage infections of both human *T. brucei* subspecies. However, after stage III clinical trials, DB289 was abandoned due to high nephrotoxicity. Despite this, DB289 has spurred the development of other diamidine derivative drugs (Kennedy, 2013).

Benzoxaborole drugs, such as SCYX-7158, are capable of crossing the blood-brain barrier *in vivo* and can clear infection of both *T. brucei* subspecies. This drug can be administered orally and also has a long half-life, allowing it to be used as a single dose. SCYX-7158 successfully completed stage I clinical trials and has been approved to move forward with stage II/III clinical trials (Jacobs et al., 2011). While no specific proteins or enzyme have yet been shown to play a role in the method of action of benzoxaboroles, evidence strongly suggests that drugs impede upon the ability of *T. brucei* to properly metabolize methionine (Steketee et al., 2018).

Fexinidazole, a nitroimidazole compound which is effective against both parasites and CNS disease, is safe and well tolerated in early studies. A recent study by Mesu et al. (2018), has reported that stage II/III clinical trials of fexinidazole against HAT have been completed and showed that oral fexinidazole is effective and safe for the treatment of *T. b*. gambiense infection compared with nifurtomox-eflornithine combination therapy in late-stage HAT patients. No method of action for nitroimidazoles has yet been elucidated, though it is known to be a substrate for a type I nitro-reductase and is theorized to function *in vivo* as a pro-drug (Wyllie et al., 2016; Papadopoulou et al., 2017).

Tyrosine kinase inhibitors, such as lapatinib and a few of its derivatives have shown some activity in controlling *T. brucei* infection (Behera et al., 2014; Woodring et al., 2015). Lapatanib mediates antiparasitic activity against trypanosomes by inhibiting four separate protein kinases leading to changes in flagellar topology and an inhibition in the parasite endocytosis. These four kinases have been termed TbLBPK 1-4 and their interruption results in the dephosphorylation of BILBO-1, kinesins, and Rab in *T. brucei* (Guyett et al., 2017).

PI3K/mTOR inhibitors, such as NVP-BEZ235 have efficacy in combating *T. brucei* infection using a mouse model (Diaz-Gonzalez et al., 2011). Through inhibition of several kinase cascades necessary for bloodstream trypanosomes to thrive, effects are seen in the stability of parasite flagellum, in the ability of trypanosomes to mount a proper stress response to complement-mediated and osmotic-lysis, and in endocytosis (Seixas et al., 2014; Fernandez-Cortes et al., 2017).

Arginase Inhibitors, namely *S*-(2-boronoethyl)-L-cysteine (BEC), have been shown to reduce the parasitic burden both *in vivo* and *in vitro*. By blocking Arginase-1 and reducing available growth factors released by macrophages, proliferation of parasites is reduced. *Tb*KHC1 has also been identified as a candidate protein involved with this immunomodulation (Nzoumbou-Boko et al., 2017). Furthermore, it has been shown that preventing the function of Arginase-1 results in the prevention of myeloid-derived suppressor cells inhibiting CD4^+^ T-cell proliferation, as well as the production of IFN-γ, both of which aid in suppression of trypanosomes (Onyilagha et al., 2018).

Phenolic or flavonoid compounds are a group of plant derived antioxidants which have shown some trypanocidal effects *in vitro* and include the compounds curcumin, gallic acid, quercetin, and resveratrol. Curcumin in particular has been shown to have immunomodulatory effects on the host, preventing damage caused by the generation of ROS (Wolkmer et al., 2013). Gallic acid and quercetin both have also been shown to have prooxidant effects, leading to a direct trypanocidal effects by generation of excess ROS while also maintaining the host protective antioxidant effects (Baldim et al., 2017). Several of these compounds have also been identified as having inhibitory effects against *T. brucei* RNA triphosphatase (Smith et al., 2016). Few *in vivo* studies have been carried out in relation to these plant derived phenolic or flavonoid antioxidant compounds, making this a target area of interest for future research.

Antioxidant vitamins, in particular vitamin C, but also vitamins E and A, have protective effects for the host during *T. brucei* infection through *in vivo* studies. These vitamins are presumed to function as antioxidant to reduce parasitemia and largely reduce organ damage associated with *T. brucei* infections (Ibrahim et al., 2016). Furthermore, vitamin C has been shown to potentiate the trypanocidal effects of diminazene aceturate in a co-administration trial (Chekwube et al., 2014). More recently, vitamin D3 has shown some efficacy in protecting the host during *T. brucei* infection, however the mechanism of action is not yet known (Jamal et al., 2016).

Chelating compounds have myriad positive effects for the host including anti-inflammatory and antioxidant properties. 1,8-naphthyridine derivative compounds are particularly of interest, as they have been shown to possess anti-trypanosomal activity via their ability to chelate Zn^2+^, Cu^2+^ and Fe^2+^ which are necessary for trypanosomes to thrive in the host (Wall et al., 2018). Thiosemicarbazone has also shown to have some efficacy in ridding a host of trypanosomes through chelation of iron (Ellis et al., 2015).

Presently, no host-targeted drugs or vaccines are described for the treatment or prevention of HAT. The presence of VSGs and the parasites’ ability to undergo antigenic variation represent a major challenge for vaccine discovery and unfortunately there has been no development of drugs to target the VSG gene switching mechanism. Potential host-targeting drugs include those affecting host immunomodulation and those which can affect a polarized shift toward a Th1 response, while increasing the production of IFN-γ to drive the clearance of the parasites before they are able to invade the CNS. We summarized the compounds in this section and their targets in Table 3.

**Table 3:** Host-targeted and anti-parasitic therapeutics for HAT.

Host-targeted drug	Classification	Mode of action	Reference
Pafuramidine (DB289)	Diamidine derivative	Interferes with the nuclear mechanisms, inhibiting synthesis of DNA, RNA	Kennedy, 2013
Acoziborole (SCYX-7158)	Benzoxaborole drug	Negatively impacts methionine metabolism	Jacobs et al., 2011; Steketee et al., 2018
Fexinidazole	Nitroimidazole compound	Nitroreductase substrate pro-drug	Wyllie et al., 2016; Papadopoulou et al., 2017; Mesu et al., 2018
Dactolisib (NVP-BEZ235)	Kinase inhibitor	PI3K/mTOR inhibitors	Diaz-Gonzalez et al., 2011; Seixas et al., 2014; Fernandez-Cortes et al., 2017
Lapatinib	Kinase Inhibitor	TbLBPK 1-4 inhibition	Guyett et al., 2017
*S*-(2-boronoethyl)-L-cysteine (BEC)	Arginase inhibitor	Reduction in quantity of growth factors available for trypanosomes	Nzoumbou-Boko et al., 2017; Onyilagha et al., 2018
Curcumin, gallic acid, quercetin, resveratrol	Phenolic or flavonoid compounds	Increases oxidative stress against the parasites, while offering oxidative protection against the host	Wolkmer et al., 2013; Smith et al., 2016; Baldim et al., 2017
Vitamins C, A, E, and D3	Antioxidant vitamins	Enhances host immune function, protects host from oxidative damage	Chekwube et al., 2014; Ibrahim et al., 2016; Jamal et al., 2016
Naphthyridine derivatives, thiosemicarbazone	Chelating compounds	Antioxidant and anti-inflammatory properties, transition metal chelation	Ellis et al., 2015; Wall et al., 2018

## Conclusion

Leishmaniasis, Chagas disease and HAT cause the highest number of deaths amongst all NTDs (Hotez et al., 2007). One of the major confounding issues to eliminating these NTDs is that they are often present in small endemic areas; they have relatively limited global disease burdens and are effectively ignored by the community at large. This is further compounded in that these endemic areas are often subject to political and military turmoil, in areas which lack infrastructural support or effective health care systems. Finally, climate change has also had an impact on the spread of NTDs, as the warmer weather increases the development of insect vectors, which escalates the transmission of these diseases (Hotez, 2017).

Because these infections are difficult to eradicate, it is imperative that more attention is directed toward finding preventive and curative therapeutics to control their spread. There are currently no prophylactic vaccines for the above-mentioned protozoan diseases and the available treatments are antiquated and have significant toxicities. Development of novel agents or utilization of existing host-targeted therapeutics is a promising avenue for the treatment of protozoan NTDs. Despite the numerous advances in the immunology and cell biology fields, we are still far from eradicating these diseases. We believe that moving forward it will be crucial to allocate more funds toward pre-clinical and especially clinical research focused on developing and testing new host-targeted therapeutics for NTDs. Unfortunately, many NTDs affect remote and rural areas of low-and middle income countries (LMICs) with limited resources, infrastructures, and medical personnel. In these areas it is challenging to follow the Good Clinical Practices (GCPs) outlined by WHO and International Conference of Harmonization (ICH) to conduct meaningful clinical trials (Boelaert and Consortium, 2016; Ravinetto et al., 2016). We hope that raising awareness about NTDs and their burden will fuel the already ongoing mobilization of resources on a global scale to aid the development of more infrastructures to conduct clinical trials as well as screening and treatment in endemic areas.

## Author Contributions

SV, BJ, GV, NR, GH, and OH reviewed the literature and wrote sections and first draft of the manuscript. AS and BM contributed to conception of the manuscript, wrote sections of the manuscript and revised the manuscript. All authors read and approved the submitted version.

## Conflict of Interest Statement

The authors declare that the research was conducted in the absence of any commercial or financial relationships that could be construed as a potential conflict of interest.
